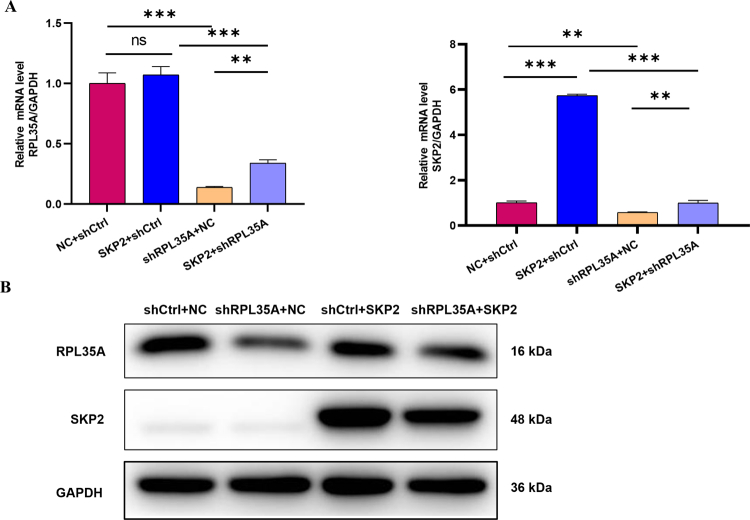# RPL35A drives aerobic glycolysis and tumorigenesis by facilitating MYC-mediated *SKP2* transcription

**DOI:** 10.1016/j.jbc.2025.110944

**Published:** 2025-11-13

**Authors:** Liqin Yan, Yifei Chen, Dawei Cao, Mingxia Hou, Ting Xue, Xinri Zhang

**Affiliations:** 1 The First College of Clinical Medicine, Shanxi Medical University, Taiyuan, China; 2 The Geriatric Medicine Department of Shanxi Provincial People Hospital, Shanxi Medical University, Taiyuan, China; 3 Department of Respiratory Medicine, Shanxi Province Cancer Hospital/Shanxi Hospital Affiliated to Cancer Hospital, Chinese Academy of Medical Sciences/Cancer Hospital Affiliated to Shanxi Medical University, Taiyuan, China; 4 NHC Key Laboratory of Pneumoconiosis, Shanxi Key Laboratory of Respiratory Diseases, Department of Pulmonary and Critical Care Medicine, The First Hospital of Shanxi Medical University, Taiyuan, China

**Keywords:** lung cancer, RPL35A, MYC, SKP2, aerobic glycolysis

## Abstract

As a ribosomal protein, RPL35A participates in diverse cellular processes, yet its role in lung cancer remains unclear. Here, we demonstrate that RPL35A is upregulated in lung cancer tissues and correlates with poor patient prognosis. Functional studies show that RPL35A overexpression enhances the proliferation, migration, and invasion of lung cancer cells while suppressing apoptosis; conversely, RPL35A knockdown produces opposing effects. *In vivo* xenograft models confirm that RPL35A depletion significantly inhibits tumor growth. Mechanistically, RPL35A directly interacts with the oncoprotein MYC through its N-terminal domain (amino acids 2–22), facilitating MYC nuclear translocation and recruitment to the SKP2 promoter. This interaction drives SKP2 expression, leading to activation of aerobic glycolysis, as evidenced by increased glucose uptake, lactate production, and extracellular acidification rate. Deletion of the 2 to 22 residue motif (RPL35A-Δ1) abolishes MYC binding and fails to restore SKP2 expression or glycolytic activity in rescue experiments. Furthermore, the oncogenic effects of RPL35A are abrogated by glycolysis inhibition, confirming metabolic reprogramming as a key downstream effector. Collectively, our findings suggest that RPL35A may serve as a valuable prognostic biomarker for lung squamous cell carcinoma (LUSC) patients and a promising therapeutic target.

Lung cancer, which primarily originates from epithelial cells of the bronchial mucosa, is one of the most common malignancies worldwide. It ranks second in global incidence and remains the leading cause of cancer-related mortality (1). With a 5-year survival rate of only approximately 19%, lung cancer poses a severe threat to human health (2).Over two-thirds of patients present with lymph node involvement or distant metastasis at diagnosis (3, 4), attributable to the disease’s insidious onset and rapid progression. These factors frequently result in late-stage diagnosis and missed opportunities for surgical intervention. Although new therapeutic modalities, including both surgical and non-surgical approaches, have been developed, limitations such as drug resistance and suboptimal efficacy in treating metastatic disease persist (5). These factors collectively contribute to the extremely poor prognosis of lung cancer patients. Therefore, there is an urgent need to identify new therapeutic targets and develop more effective small-molecule targeted drugs to improve treatment outcomes and patient prognosis.

Ribosomal proteins (RPs) are essential components of the ribosome, playing crucial roles in ribosome biosynthesis, protein translation, and various cellular processes such as cell proliferation, apoptosis, migration, and cell cycle regulation (6, 7). Beyond their canonical ribosomal role, ribosomal proteins (RPs) exhibit diverse extra-ribosomal functions. These include modulating key cellular stress-response pathways, both p53-dependent and p53-independent, which can ultimately induce cell cycle arrest and apoptosis (8). RPL35A, a component of the L35AE family of ribosomal proteins, is encoded by a gene located on human chromosome 3q29–qter (9). As an essential component of the 60S ribosomal subunit, RPL35A depletion has been shown to significantly inhibit the growth of various cancer cells (10). Beyond its role in ribosomal biogenesis, RPL35A has been implicated in Diamond–Blackfan anemia (DBA), a ribosomopathy associated with heightened cancer susceptibility (11, 12). Furthermore, elevated RPL35A expression has been reported in hepatocellular carcinoma (HCC) (13), and it has been identified as a key promoter of gastric cancer progression as well as a potential biomarker for tumor angiogenesis (14).However, the expression patterns, functional roles, and molecular mechanisms of RPL35A in lung cancer remain largely elusive. This study therefore aims to elucidate the functional role and mechanistic basis of RPL35A in lung cancer progression, with the goal of providing a theoretical rationale for its potential as a novel therapeutic target.

## Results

### RPL35A is highly expressed in lung cancer and correlates with poor prognosis

**Figure 1:**
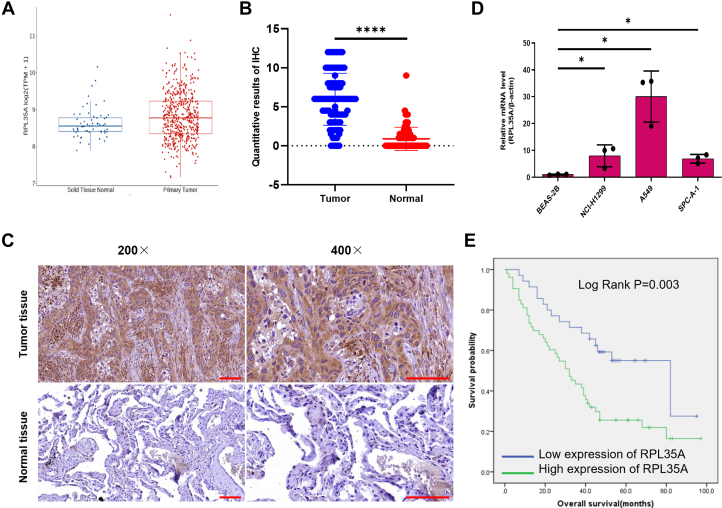
**RPL35A is significantly up-regulated in lung cancer.***A*, expression levels of *RPL35A* in tumor and normal tissues were obtained from TCGA datasets (GDC: https://portal.gdc.cancer.gov/) and analyzed using the Wilcoxon test in R. *B*, quantitative analysis of RPL35A expression in lung cancer tissues (n = 88) and adjacent normal tissues (n = 77) based on immunohistochemistry (IHC) staining. *C*, IHC images were utilized to reveal the expression of RPL35A in lung cancer tissues compared to normal tissues. The *red scale bar* represents 100 μm. *D*, the expression level of RPL35A in lung cancer cell lines (A549, NCI-H1299, SPC-A-1) and normal pulmonary epithelial cells (BEAS-2B) was examined by qPCR. *E*, Kaplan-Meier survival analysis indicated that increased RPL35A expression is associated with shorter survival duration. Statistical significance: ∗*p* < 0.05, *p* < 0.01, ∗*p* < 0.001.

Analysis of RNA sequencing data from 516 lung adenocarcinoma and 59 normal tissue samples in The Cancer Genome Atlas (TCGA) revealed significantly higher *RPL35A* expression in tumor tissues compared to normal controls (Fig. 1*A*). To validate these findings, we performed Immunohistochemistry IHC staining on a cohort of 88 lung cancer tissues and 77 paired adjacent non-tumor tissues. Using a scoring system based on staining intensity and extent, an IHC score >6 was defined as high expression, while a score ≤6 was considered low expression (Fig. 1*B*). IHC results consistently demonstrated elevated RPL35A expression in tumor tissues compared to adjacent non-tumor tissues (Table 1 and Fig. 1*C*). Consistent with these findings, RPL35A expression was also markedly elevated in lung cancer cells (Fig. 1*D*).

**Table 1:** Expression patterns in lung cancer tissues and para-carcinoma tissues revealed in immunohistochemistry analysis

RPL35A expression	Tumor tissue		Normal tissue		*p* value
	Cases	Percentage	Cases	Percentage	
Low	35	39.8%	76	98.7%	*p* < 0.001
High	53	60.2%	1	1.3%	

To evaluate the clinical significance of RPL35A expression, we analyzed its association with pathological features. The Mann–Whitney *U* test showed that higher RPL35A expression was significantly associated with advanced pathological grade (Table 2). This correlation was further confirmed by Spearman’s rank correlation analysis (Table 3), suggesting that elevated RPL35A expression is linked to more aggressive disease.

**Table 2:** Relationship between RPL35A expression and tumor characteristics in patients with lung cancer

Features	Patients number	RPL35A expression		*p* value
		low	High	
All patients	88	35	53	
Age				0.965
≤63 years	45	18	27	
>63 years	43	17	26	
Gender				0.824
Male	49	20	29	
Female	39	15	24	
Grade				*p* < 0.001
I	4	3	1	
II	43	24	19	
III	41	8	33	
Metastasis				0.766
M0	86	34	52	
M1	2	1	1

**Table 3:** Relationship between RPL35A expression and tumor characteristics in patients with lung cancer

Variable	Description	Statistical outcome
Grade	Spearman correlation	0.395
	Signification (double-tailed)	*p* < 0.001
	Patients number	88

Kaplan–Meier survival analysis, stratifying patients based on IHC scores (high: >6 vs. low: ≤6), demonstrated that high RPL35A expression was significantly associated with shorter overall survival (hazard ratio [HR] = 1.82; 95% confidence interval [CI]: 1.21 to 2.74; *p* = 0.003; Fig. 1*E*). These results indicate that RPL35A overexpression is a predictor of poor prognosis in patients with lung cancer (Fig. 1*E*).

### RPL35A promotes proliferation and migration and inhibits apoptosis in lung cancer cells

**Figure 2:**
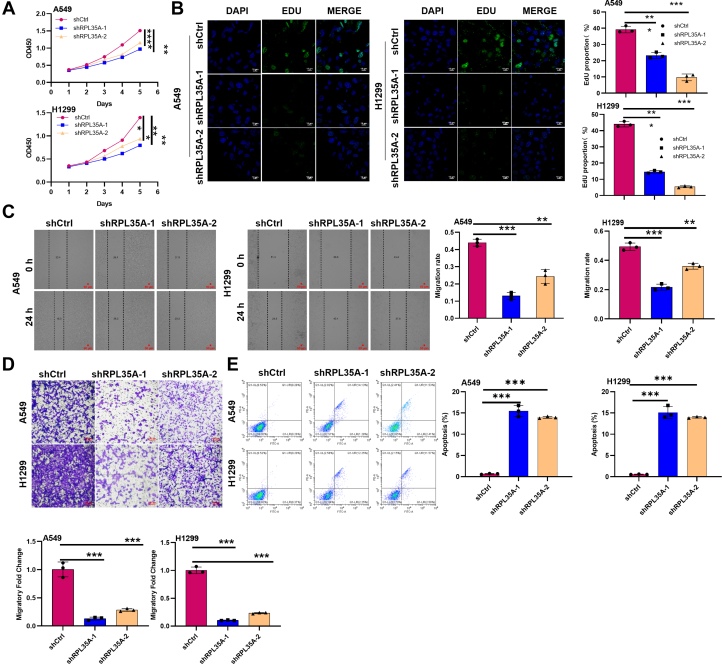
**Knockdown of RPL35A inhibits proliferation and migration, and promotes apoptosis in lung cancer cells.***A*, cell proliferation was assessed by CCK-8 assay in A549 and H1299 cells following RPL35A knockdown. *B*, EDU assay was performed to evaluate the effect of RPL35A knockdown on cell proliferation. The *white scale bar* represents 10 μm. *C*, wound healing assay was used to assess the migratory capacity of A549 and H1299 cells after RPL35A silencing. The *red scale bar* represents 50 μm. *D*, Transwell assay was applied to further evaluate the migration ability of RPL35A-knockdown cells. The *red scale bar* represents 100 μm. *E*, flow cytometry analysis using Annexin V-FITC/PI staining revealed increased apoptosis in RPL35A-deficient cells. Data are presented as mean ± SD. Statistical significance: *p* < 0.01, ∗*p* < 0.001.

**Figure S1:**
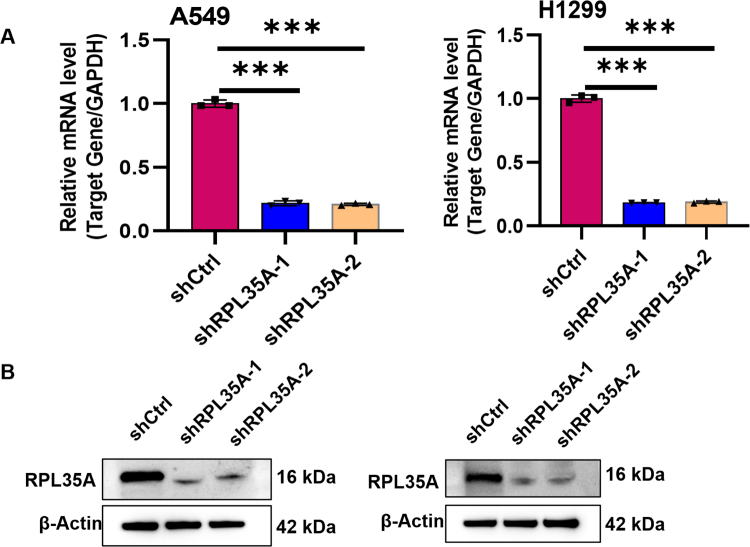


To investigate the functional role of RPL35A in lung cancer progression, we first established stable knockdown models in A549 and NCI-H1299 cell lines using two specific shRNA sequences targeting *RPL35A*. The efficiency of knockdown was successfully validated at both the mRNA and protein levels by quantitative PCR (qPCR) and Western blot (WB) analysis (Fig. S1, *A* and *B*). Cell counting assays demonstrated that knockdown of RPL35A suppressed the proliferation of both lung cancer cell lines (Fig. 2*A*). This finding was further validated by EdU incorporation assays, which showed a marked reduction in DNA synthesis (Fig. 2*B*). Wound healing and Transwell migration assays consistently revealed that RPL35A knockdown markedly impaired the migratory capacity of the cancer cells (Fig. 2, *C* and *D*). Moreover, flow cytometric analysis indicated a substantial increase in apoptosis following RPL35A knockdown (Fig. 2*E*). Together, these loss-of-function experiments demonstrate that RPL35A is required for the proliferation, migration, and survival of lung cancer cells.

**Figure 3:**
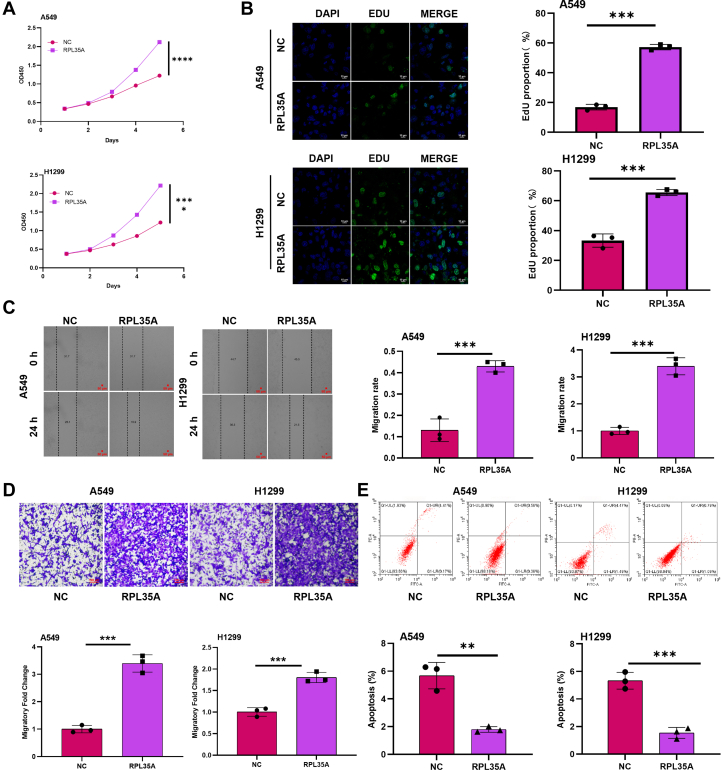
**Overexpression of RPL35A promotes proliferation and migration, and suppresses apoptosis in lung cancer cells.***A*, CCK-8 assay was used to detect cell proliferation in A549 and H1299 cells upon RPL35A overexpression. *B*, EDU assay was performed to evaluate the proliferative capacity of RPL35A-overexpressing cells. The *white scale bar* represents 10 μm. *C*, wound-healing assay was conducted to assess cell migration following RPL35A overexpression. The *red scale bar* represents 50 μm. *D*, Transwell assay was employed to further confirm the enhanced migratory ability induced by RPL35A overexpression. The red scale bar represents 100 μm. *E*, flow cytometry analysis with Annexin V-FITC/PI staining revealed a significant decrease in apoptosis in RPL35A-overexpressing cells. Data are presented as mean ± SD. Statistical significance: *p* < 0.01, ∗*p* < 0.001.

To complement these findings, we generated a stable RPL35A-overexpressing NCI-H1299 cell line using a lentiviral expression system, with successful overexpression confirmed by qPCR and Western blot (S2A, S2B). Consistent with the knockdown results, RPL35A overexpression significantly enhanced cell proliferation, as evidenced by both cell counting and EdU incorporation assays (Fig. 3, *A* and *B*). Wound healing and Transwell assays indicated that RPL35A overexpression enhanced cell migration (Fig. 3, *C* and *D*). Notably, flow cytometric analysis demonstrated a significant decrease in apoptosis in RPL35A-overexpressing cells (Fig. 3*E*). These gain-of-function data confirm that RPL35A is sufficient to drive oncogenic phenotypes in lung cancer cells.

Collectively, these results establish RPL35A as a critical regulator of malignant behaviors in lung cancer, promoting tumor cell proliferation and migration while inhibiting apoptosis.

### RPL35A knockdown inhibits lung cancer progression by decreasing glycolytic activity

**Figure 4:**
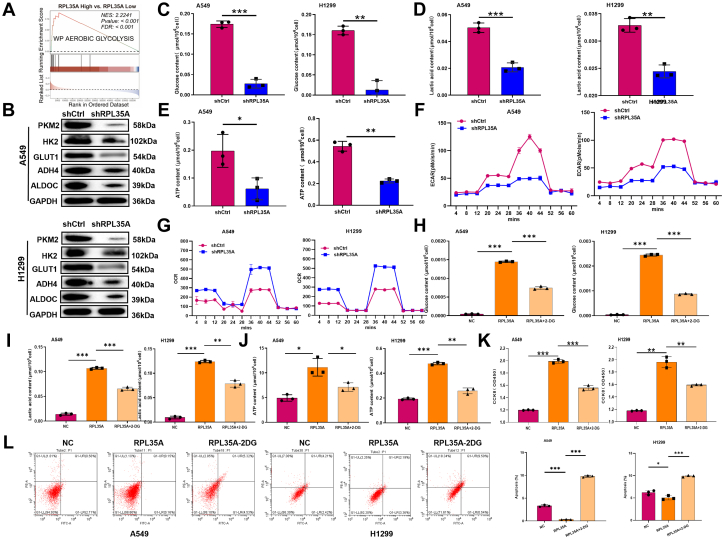
**RPL35A knockdown suppresses glycolytic activity in lung cancer cells.***A*, gene set enrichment analysis (GSEA) revealed a positive correlation between *RPL35A* expression and aerobic glycolysis. *B*, Western blot analysis showed downregulation of key glycolytic enzymes (PKM2, HK2, GLUT1, ADH4, ALDOC) in A549 and H1299 cells following RPL35A knockdown. *C–F*, glucose consumption (*C*), lactate production (*D*), ATP levels (*E*), and extracellular acidification rate (ECAR), and (*F*) were significantly reduced upon RPL35A silencing, indicating impaired glycolysis. *G*, oxygen consumption rate (OCR) was increased after RPL35A knockdown, suggesting a shift toward oxidative phosphorylation. *H–J*, in RPL35A-overexpressing cells, treatment with the glycolysis inhibitor 2-DG reduced glucose uptake (*H*), lactate production (*I*), and ATP levels (*J*). *K*, 2-DG treatment attenuated the enhanced cell proliferation induced by RPL35A overexpression. *L*, 2-DG enhanced apoptosis in RPL35A-overexpressing cells. Data are presented as mean ± SD. Statistical significance: ∗*p* < 0.05, *p* < 0.01, ∗*p* < 0.001.

The GSEA analysis shows the significant enrichment of genes involved in aerobic glycolysis in lung cancer samples with high *RPL35A* expression compared to those with low *RPL35A* expression (Fig. 4*A*), suggesting a potential link between RPL35A and metabolic reprogramming. To validate this GSEA result, we assessed key indicators of aerobic glycolysis in lung cancer cells following RPL35A knockdown. The results demonstrated marked reductions in the expression levels of critical aerobic glycolytic enzymes including PKM2, HK2, GLUT1, ADH4, and ALDOC (Fig. 4*B*). Notably, aerobic glycolytic activity was significantly suppressed in RPL35A-knockdown lung cancer cells, as demonstrated by reduced glucose uptake, ATP production, and lactate secretion, along with decreased extracellular acidification rate (ECAR) and increased oxygen consumption rate (OCR) (Fig. 4, *C*–*G*), indicating a metabolic shift from glycolysis toward oxidative phosphorylation. Furthermore, to determine whether the oncogenic effects of RPL35A overexpression depend on glycolytic activity, we treated RPL35A-overexpressing lung cancer cells with the glycolytic inhibitor 2-DG. As expected, 2-DG treatment effectively reduced glucose consumption, lactate production, and intracellular ATP levels (Fig. 4, *H*–*J*). Importantly, inhibition of glycolysis not only reversed the hyperproliferative phenotype but also induced significant apoptosis (Fig. 4, *K* and *L*). Collectively, these findings indicate that RPL35A drives lung cancer progression through enhancing aerobic glycolysis.

### RPL35A promotes the transcriptional activation of *SKP2* by facilitating MYC nuclear translocation and DNA binding

**Figure 5:**
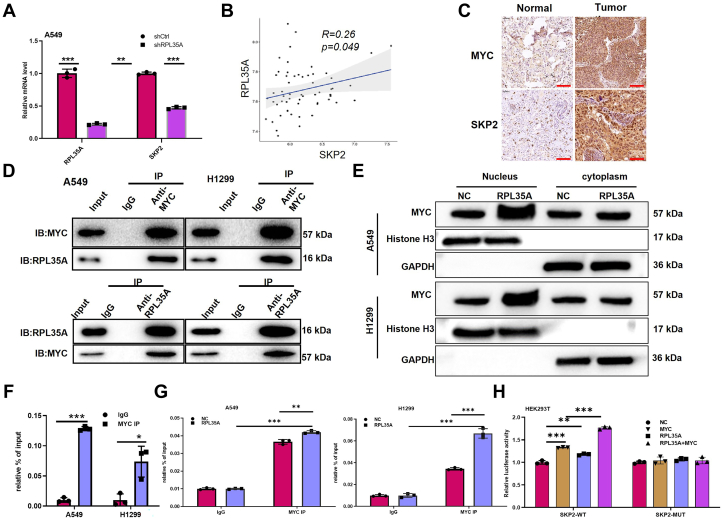
**RPL35A regulates SKP2 expression through MYC.***A*, SKP2 expression was significantly decreased in RPL35A-knockdown A549 cells, as determined by qPCR. *B*, a positive correlation between RPL35A and SKP2 expression levels was observed in lung cancer samples. *C*, IHC analysis revealed co-expression of MYC and SKP2 in human lung cancer tissues. The *red scale bar* represents 100 μm. *D*, co-immunoprecipitation (Co-IP) assays demonstrated a direct physical interaction between RPL35A and MYC in lung cancer cells. *E*, overexpression of RPL35A promoted nuclear translocation of MYC. *F*, chromatin immunoprecipitation followed by qPCR (ChIP-qPCR) confirmed that the transcription factor MYC binds to the promoter region of SKP2. *G*, RPL35A overexpression enhanced MYC binding to the SKP2 promoter, as shown by ChIP-qPCR. *H*, Luciferase reporter assays indicated that RPL35A promotes MYC-mediated transcriptional activation of the SKP2 promoter. Data are presented as mean ± SD. Statistical significance: ∗*p* < 0.05, *p* < 0.01, ∗*p* < 0.001.

**Figure S3:**
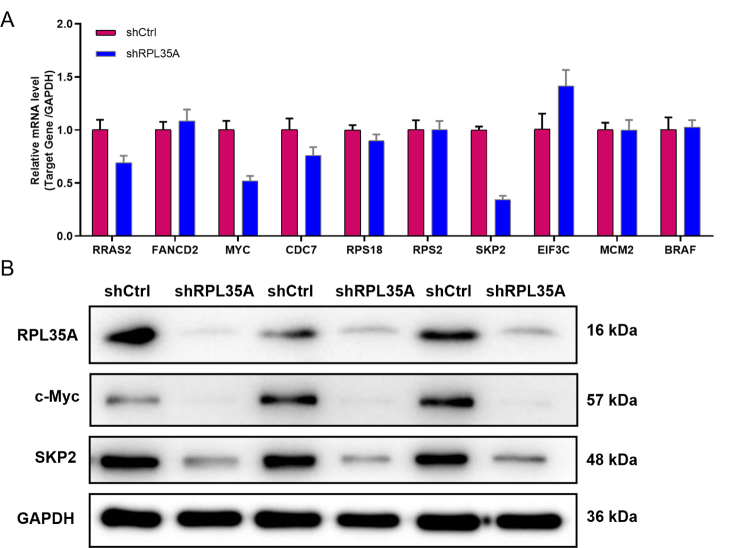


To further investigate the molecular mechanisms by which RPL35A contributes to lung cancer progression, we performed Affymetrix microarray analysis in RPL35A knockdown and control cells. The results showed that RPL35A knockdown significantly downregulated the expression of multiple genes, including *MYC*, *FANCD2*, and *SKP2*, among others (Fig. S3*A*). Of these, *SKP2* was among the most notably affected (Fig. 5*A*). Consistent with these findings, analysis of lung cancer datasets from the GEO database revealed a strong positive correlation between *RPL35A* and *SKP2* expression (Fig. 5*B*). IHC staining of paired adjacent non-tumor and lung cancer tissue samples from patients showed markedly elevated expression of MYC and SKP2 in cancerous tissues (Fig. 5*C*). It was also confirmed that RPL35A knockdown led to reduced expression of both MYC and SKP2 (Fig. S3*B*). Integrating transcription factor regulatory information from the GTRD database, we hypothesized that MYC may transcriptionally regulate its downstream target gene *SKP2*.

Given that MYC is a well-established transcriptional regulator of SKP2, as supported by regulatory annotations in the GTRD database, we hypothesized that RPL35A may modulate SKP2 expression through MYC-dependent transcriptional activation. To test this, we first examined whether RPL35A interacts with MYC. Bidirectional co-immunoprecipitation (Co-IP) assays confirmed a physical interaction between RPL35A and MYC in lung cancer cells (Fig. 5*D*). Subsequent fractionation of nuclear and cytoplasmic proteins from lung cancer cells demonstrated that upregulation of RPL35A significantly enhanced the nuclear accumulation of MYC (Fig. 5*E*), suggesting that RPL35A facilitate MYC’s nuclear translocation. Chromatin immunoprecipitation (ChIP) assays further verified that MYC binds to the promoter region of *SKP2* (Fig. 5*F*), supporting its role as a transcriptional regulator of *SKP2*. Moreover, ChIP-qPCR analysis in RPL35A-overexpressing and control lung cancer cells indicated that RPL35A overexpression markedly strengthens the recruitment of MYC to the *SKP2* promoter (Fig. 5*G*). In addition, dual-luciferase reporter assays in HEK293T cells transfected with either wild-type or mutant *SKP2* promoter constructs showed that co-overexpression of RPL35A and MYC increased transcriptional activity specifically from the wild-type—but not the mutant—promoter (Fig. 5*H*).

In summary, this study reveals that RPL35A is highly expressed in lung cancer and promotes *SKP2* transcription by facilitating MYC nuclear translocation and enhancing its binding to the *SKP2* promoter. These findings provide novel mechanistic insights into the role of RPL35A in lung cancer progression.

### RPL35A regulates aerobic glycolysis and phenotypic progression through SKP2

**Figure 6:**
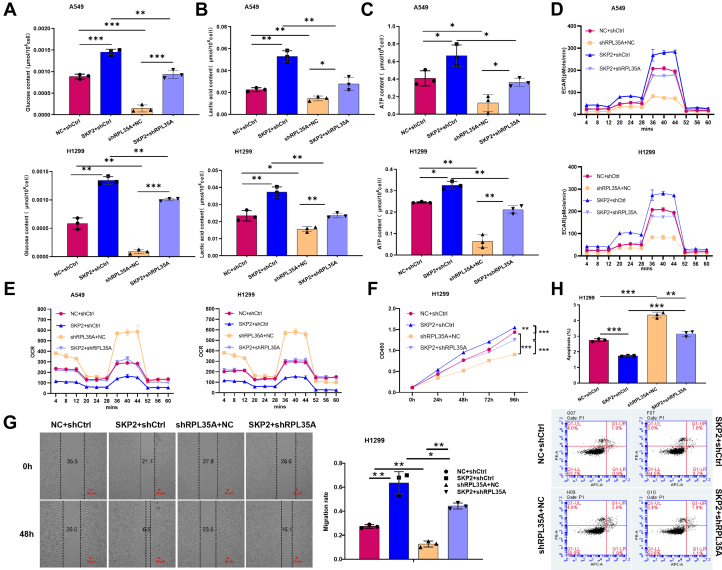
**SKP2 overexpression reverses the effects of RPL35A knockdown on aerobic glycolysis, cell proliferation, migration, and apoptosis.***A*, overexpression of SKP2 reversed the reduction in glucose uptake induced by RPL35A knockdown. *B*, lactate production, which was decreased following RPL35A knockdown, was restored by SKP2 overexpression. *C*, ATP levels, reduced due to RPL35A knockdown, were restored by SKP2 overexpression. *D*, extracellular acidification rate (ECAR), depleted upon RPL35A knockdown, was replenished by SKP2 overexpression. *E*, oxygen consumption rate (OCR), elevated after RPL35A knockdown, was depressed by SKP2 overexpression. *F*, cell proliferation, inhibited by RPL35A knockdown, was restored by SKP2 overexpression. *G*, cell migration, impaired by RPL35A knockdown, was rescued by SKP2 overexpression. The *red scale bar* represents 50 μm. *H*, apoptosis, increased by RPL35A knockdown, was attenuated by SKP2 overexpression. Data are presented as mean ± SD. Statistical significance: ∗*p* < 0.05, *p* < 0.01, ∗*p* < 0.001.

Previous studies have demonstrated that elevated expression of SKP2 enhances glycolysis and facilitates tumor progression by promoting the degradation of isocitrate dehydrogenase 1 (IDH1) (15, 16, 17). This finding prompted us to investigate whether RPL35A-mediated upregulation of SKP2 expression could enhance aerobic glycolysis and promote tumor progression in lung cancer cells. To test this hypothesis, we generated a rescue model in which SKP2 was overexpressed in RPL35A-knockdown lung cancer cells (Fig. S4, *A* and *B*). Functional assays revealed that SKP2 overexpression effectively restored the suppression of aerobic glycolysis induced by RPL35A knockdown, as evidenced by increased glucose uptake, lactate production, ATP levels, ECAR and decreased OCR (Fig. 6, *A*–*E*). Moreover, SKP2 overexpression significantly reversed the inhibitory effects of RPL35A knockdown on cell proliferation and migration (Fig. 6, *F* and *G*). Additionally, SKP2 overexpression markedly attenuated the apoptosis resulting from RPL35A knockdown (Fig. 6*H*). Together, these findings indicate that co-targeting RPL35A and SKP2 may represent a promising strategy to suppress aerobic glycolysis and mitigate malignant phenotypes in lung cancer cells.

### The 2 to 22 amino acid residues of RPL35A mediate MYC interaction, glycolysis, and tumor progression

**Figure 7:**
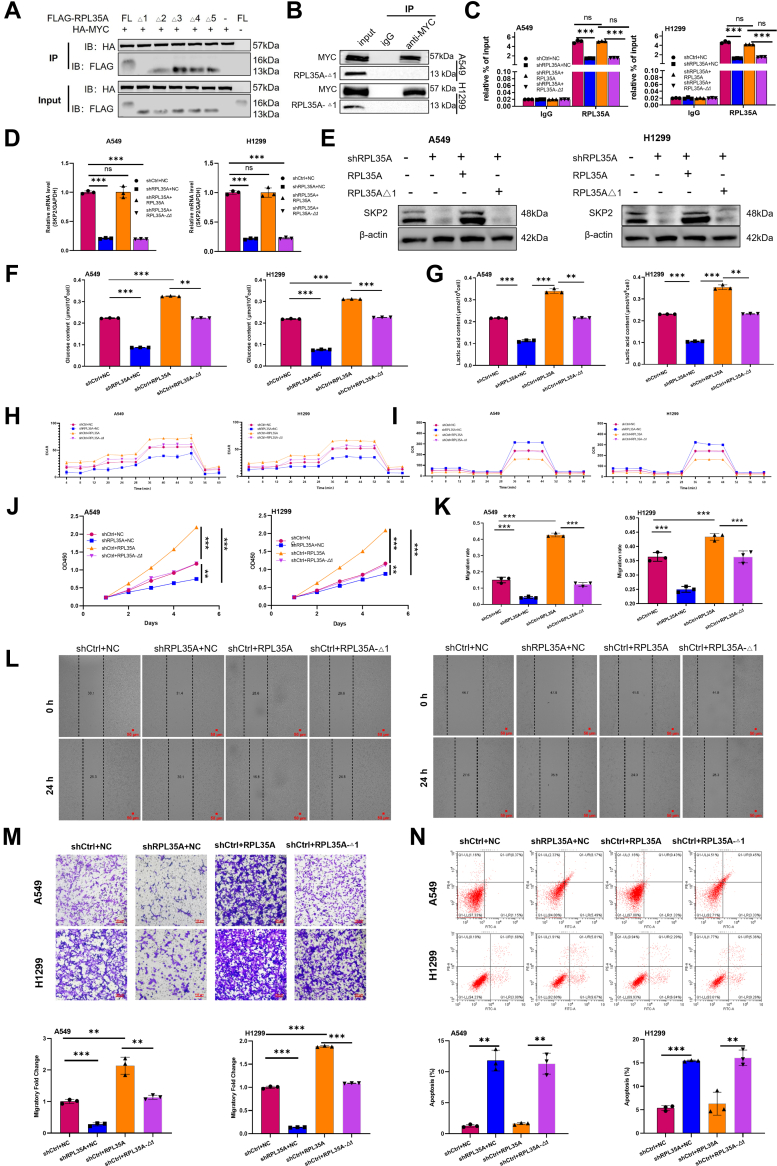
**The 2 to 22 amino acid residues of RPL35A are essential for MYC interaction, glycolytic activation, and tumor progression.***A*, co-immunoprecipitation (Co-IP) assays using serial truncation mutants of RPL35A (Δ1: aa 2–22 deletion; Δ2: aa 23–44 deletion; Δ3: aa 45–66 deletion; Δ4: aa 67–88 deletion; Δ5: aa 89–110 deletion) revealed that the N-terminal 2 to 22 domain is required for binding to MYC. *B*, Co-IP validation confirmed that the RPL35A-Δ1 mutant, but not the wild-type protein, fails to interact with MYC. *C*, chromatin immunoprecipitation (ChIP) assays showed that overexpression of wild-type RPL35A promotes MYC binding to the SKP2 promoter, whereas the Δ1 mutant does not. *D*, qPCR analysis demonstrated that the Δ1 mutant is unable to restore *SKP2* mRNA expression in RPL35A-knockdown cells. *E*, Western blot analysis confirmed that the Δ1 mutant fails to rescue SKP2 protein expression. *F–I*, functional assays showed that wild-type RPL35A enhances glucose uptake (*F*), lactate production (*G*), and extracellular acidification rate (ECAR) (*H*), while reducing oxygen consumption rate (OCR) (*I*); these effects are abolished in the Δ1 mutant. *J*, cell proliferation was promoted by wild-type RPL35A, but not by the Δ1 mutant. *K–L*, Wound healing assays indicated that wild-type RPL35A enhances cell migration, whereas the Δ1 mutant has no effect. The *red scale bar* represents 50 μm. *M*, Transwell assays revealed that wild-type RPL35A increases cell invasion, while the Δ1 mutant does not. The *red scale bar* represents 100 μm. *N*, flow cytometry analysis showed that wild-type RPL35A suppresses apoptosis, whereas the Δ1 mutant fails to do so. Data are presented as mean ± SD. Statistical significance: ∗*p* < 0.05, *p* < 0.01, ∗*p* < 0.001.

To identify the essential domain for the interaction between RPL35A and MYC, we performed Co-IP assays using a series of truncated constructs of RPL35A. These experiments revealed that the region encompassing amino acid residues 2 to 22 of RPL35A is required for its binding to MYC. Consistent with this, co-IP confirmed that a mutant lacking these residues (RPL35A-Δ1) failed to interact with MYC (Fig. 7, *A* and *B*).

Following RPL35A knockdown, we rescued cells by re-expressing either wild-type RPL35A or the RPL35A-Δ1 mutant. ChIP assays showed that only wild-type RPL35A restored the binding of MYC to the SKP2 promoter; the RPL35A-Δ1 had no significant effect (Fig. 7*C*). Similarly, reconstitution with wild-type RPL35A, but not the RPL35A-Δ1, rescued the expression of both SKP2 mRNA and protein (Fig. 7, *D*-–*E*), indicating that the 2 to 22 domain is essential for SKP2 regulation.

Furthermore, overexpression of the RPL35A-Δ1 did not significantly affect glucose uptake, lactate production, ECAR, or OCR (Fig. 7, *F*–*I*), suggesting that the RPL35A–MYC interaction is necessary for modulating glycolysis *via* SKP2. Finally, functional assays revealed that while wild-type RPL35A promoted cell proliferation and migration and suppressed apoptosis, the RPL35A-Δ1 mutant lost these oncogenic capabilities (Fig. 7, *J*–*N*).

In conclusion, our results demonstrate that RPL35A binds to MYC *via* its 2 to 22 amino acid residues, thereby facilitating MYC-dependent SKP2 transcription, enhancing glycolysis, and promoting tumor progression.

### RPL35A knockdown inhibits tumor formation *in vivo*

**Figure 8:**
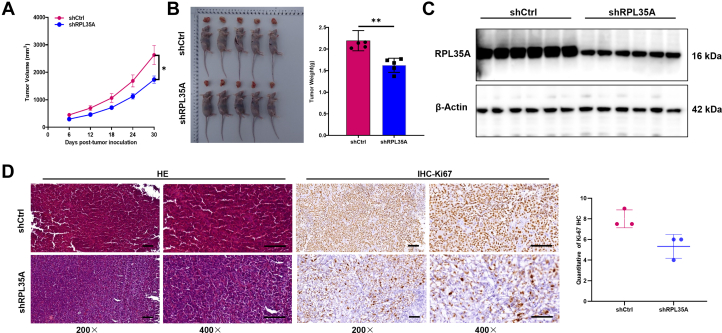
**RPL35A knockdown suppresses tumor growth *in vivo*.***A*, tumor growth curves showing reduced tumor volume in nude mice xenografted with RPL35A-knockdown A549 cells compared to control group. *B*, representative images of excised tumors and quantitative analysis of tumor weight at the endpoint. *C*, Western blot analysis confirmed the knockdown efficiency of RPL35A in harvested tumor tissues. *D*, hematoxylin and eosin (H&E) staining was performed to examine the histopathological morphology of tumor tissues. IHC was used to assess RPL35A expression and Ki-67 levels in tumor sections, indicating reduced proliferation in the knockdown group. The *black scale bar* represents 100 μm. Data are presented as mean ± SD. Statistical significance: ∗*p* < 0.05, *p* < 0.01.

To further validate the role of RPL35A in tumorigenesis, we subcutaneously injected RPL35A-knockdown or control lung cancer cells into nude mice. By observing tumor growth and monitoring tumor volume changes, we found that RPL35A knockdown significantly slowed down the tumor growth rate (Fig. 8*A*). Consistent with this observation, the final tumor weights, measured after euthanasia and tumor excision, were markedly reduced in the RPL35A-knockdown group (Fig. 8*B*). These results indicate that RPL35A knockdown attenuates tumor growth *in vivo*. Western blot analysis of the tumor tissues from each group confirmed the RPL35A expression levels, reinforcing the aforementioned findings (Fig. 8*C*). Furthermore, hematoxylin and eosin (H&E) and IHC staining of tumor sections revealed significantly reduced expression of the proliferation marker Ki67 in the RPL35A-knockdown group (Fig. 8*D*). The decreased expression of Ki67 in the RPL35A-knockdown group further supports the notion that RPL35A plays a crucial role in promoting tumor proliferation. Our *in vivo* findings demonstrated that RPL35A promotes lung cancer tumorigenesis and suggest its potential as a therapeutic target.

## Discussion

Current therapeutic strategies for lung cancer remain insufficient in effectively reducing mortality rates, highlighting the urgent need to uncover novel molecular mechanisms driving tumorigenesis. Numerous studies have emphasized the genetic heterogeneity and molecular diversity of lung cancer, which manifest through diverse dysregulated cellular pathways, distinct molecular expression profiles, and differential treatment responses (18, 19, 20). Within this complex signaling network, various genes and proteins play pivotal roles. Although specific somatic mutations—such as those in *EGFR*, *KRAS*, and EML4-ALK—have been identified, particularly in lung adenocarcinoma, they are absent in the majority of patients with lung cancer (21, 22). Thus, the identification of novel molecular targets, the development of targeted therapies, and the elucidation of their mechanisms are essential for advancing lung cancer treatment.

In this study, we report a novel finding: RPL35A is significantly upregulated in lung cancer tissues and contributes to tumor progression through the regulation of aerobic glycolysis. RPL35A has been identified as a biomarker in tumor angiogenesis (23) and exhibits elevated expression levels across multiple tumor types (24, 25). Moreover, the complex of lncNB1 and RPL35 has been shown to facilitate E2F1 protein synthesis, stabilize N-MYC, and promote N-MYC-driven tumorigenesis (26). Here, we provide the first evidence that RPL35A expression is elevated in lung cancer specimens compared to adjacent normal tissues and is correlated with patient prognosis, suggesting its role as an oncogene. This upregulation was confirmed at both the mRNA and protein levels by qRT-PCR and IHC, respectively. Functional assays revealed that RPL35A knockdown suppresses proliferation, migration, and invasion, while promoting apoptosis in lung cancer cells. Conversely, RPL35A overexpression exerts the opposite effects. These findings were further validated *in vivo*, where RPL35A knockdown led to reduced tumor size, weight, and decreased Ki67 expression.

Aerobic glycolysis, or the Warburg effect, describes the metabolic shift in cancer cells toward lactate production from glucose, even under normoxic conditions (27). Rapidly proliferating cancer cells undergo incomplete glucose metabolism, producing large amounts of lactate instead of relying on mitochondrial ATP synthesis to sustain growth. Although the complete mechanistic basis remains unclear, cells exhibiting the Warburg effect can efficiently catabolize glucose even when mitochondrial oxidative phosphorylation is functional (28). Key glycolytic enzymes such as hexokinase, phosphofructokinase, and lactate dehydrogenase have been implicated in lung cancer progression (29). Additionally, molecules including LINC01123 (30), specific amino acids (31), and sinomenine (32) have been proposed to modulate glycolysis and represent potential biomarkers or therapeutic targets. In this study, we initially identified a pro-glycolytic role of RPL35A through database analyses. Subsequent experimental evaluation revealed that RPL35A knockdown reduces glucose consumption, lactate production, ATP levels, extracellular acidification rate (ECAR), and raised oxygen consumption rate (OCR). Conversely, RPL35A overexpression enhanced these metrics and promoted proliferation while inhibiting apoptosis. The suppressive effects of glycolysis inhibitors further confirmed RPL35A as a positive regulator of aerobic glycolysis.

To elucidate the detailed mechanism by which RPL35A regulates aerobic glycolysis, we focused on SKP2, identified as a major downstream effector of RPL35A. Our research has revealed that the expression of SKP2 is increased in lung cancer tissues, and RPL35A is positively correlated with SKP2. This leads to the promotion of aerobic glycolysis and proliferation/migration of lung cancer cells, while inhibiting the apoptosis of lung cancer cells. Additionally, we propose that MYC, an established transcription activator of SKP2, participates in this regulatory process.

MYC is an oncoprotein consisting of an N-terminal transactivation domain (TAD) and a well-characterized C-terminal DNA-binding domain. The TAD (spanning amino acid residues 1–143) regulates MYC’s biological activity and is essential for MYC-mediated transcriptional activation (33). The C-terminal region contains a basic helix-loop-helix leucine zipper (bHLH-LZ) motif, which enables heterodimerization with MAX; this dimerization is required for MYC to bind to the promoters of target genes (34). The MYC oncogene family includes c-MYC, N-MYC, and L-MYC, among which c-MYC is the most extensively studied and is ubiquitously expressed during tissue development and in various tumor types. MYC exerts diverse biological functions critical for cellular metabolism (35, 36, 37). Experimental evidence has shown that MYC contributes to lung cancer progression through multiple signaling pathways, including the Wnt and PI3K/AKT pathways (38). MYC-responsive metabolic genes—such as lactate dehydrogenase A (LDHA) and ornithine decarboxylase (ODC)—encode enzymes directly involved in glycolysis (39, 40).Importantly, MYC directly binds to and activates theSKP2 promoter to regulate its expression (41, 42).

SKP2, a core component of the SKP1–Cullin1–F-box (SCF) ubiquitin E3 ligase complex, interacts directly with Skp1 and indirectly with Cullin1 and Rbx1. This assembly facilitates the recruitment of the E2 conjugase to substrate proteins, thereby enabling ubiquitin ligase activity. SKP2 plays a vital role in regulating key cellular processes—including proliferation, apoptosis, migration, and cell cycle progression—all of which contribute to tumorigenesis (43, 44, 45). Previous studies have shown that the SCF–SKP2 complex promotes Akt activation, which enhances aerobic glycolysis and sustains hyperproliferation in cancer cells (46). Correspondingly, SKP2 degradation has been reported to suppress aerobic glycolysis and inhibit colorectal cancer growth, underscoring its role in promoting glycolytic metabolism and tumor progression (47).

In this study, we demonstrated that RPL35A interacts with MYC and facilitates its nuclear translocation, thereby promoting the binding of the transcription factor MYC to the SKP2 promoter. This interaction enhances SKP2 expression, which in turn stimulates aerobic glycolysis and proliferation of lung cancer cells, while suppressing apoptosis. More detailed analyses revealed that amino acid residues 2 to 22 of RPL35A constitute the critical domain required for its interaction with MYC. Consistent with this, a deletion mutant lacking these residues (RPL35A-Δ1) failed to bind to MYC. Accordingly, rescue experiments showed that reintroduction of wild-type RPL35A, but not the Δ1 mutant, restored MYC binding to the SKP2 promoter and increased SKP2 expression. Consequently, only wild-type RPL35A enhanced glucose uptake, lactate production, extracellular acidification rate (ECAR), oxygen consumption rate (OCR), as well as promoted proliferation and migration, and inhibited apoptosis in lung cancer cells—all of which were abrogated in the Δ1 mutant group.

In summary, RPL35A is upregulated in lung cancer and correlates with poor patient survival. Mechanistically, RPL35A enhances aerobic glycolysis and promotes malignant progression of lung cancer *via* a MYC-SKP2 signaling axis. These findings highlight the potential of RPL35A as a novel therapeutic target for lung cancer intervention.

## Experimental procedures

### Bioinformatics analysis

To investigate the differential expression of RPL35A in lung adenocarcinoma (LUAD), RNA sequencing data were retrieved from The Cancer Genome Atlas (TCGA) *via* the Genomic Data Commons (GDC) Data Portal (https://portal.gdc.cancer.gov/). The dataset included transcriptomic profiles of 516 primary lung adenocarcinoma tissues and 59 adjacent non-tumor lung tissues, with 58 samples available as paired tumor–normal specimens from the same individuals. Gene-level expression quantification was performed using transcripts per million (TPM) values derived from RNA-seq data (hg38 reference genome). For downstream analysis, RPL35A mRNA expression levels were log_2_-transformed after adding a pseudo-count of 1 (log_2_[TPM + 1]) to normalize variance and stabilize dispersion across orders of magnitude.

Differential expression analysis between tumor and normal tissues was conducted using R version 4.3.1 within the RStudio environment. The Wilcoxon rank-sum test (also known as the Mann–Whitney *U* test) was employed to assess statistical significance due to the non-normal distribution of gene expression values. Benjamini–Hochberg false discovery rate (FDR) correction was applied to adjust for multiple testing, and adjusted *p*-values (q-values) < 0.05 were considered statistically significant.

### Tissue specimens

A tissue microarray comprising 88 lung tumor specimens and 77 matched adjacent normal tissues was acquired from Shanghai Xinchao Biotechnology Co, Ltd for subsequent molecular profiling. All procedures strictly adhered to the Declaration of Helsinki and institutional ethical regulations. Written informed consent was obtained from all participants providing tissue samples. Comprehensive clinicopathological data, including age, gender, histological grade, TNM stage, and metastasis status, were systematically documented. This study was approved by the Independent Ethics Committee of Xinchao Biotechnology Co, Ltd (Approval Date: 12 April 2023; Approval No.: SHYJS-CP-2304002).

### Immunohistochemistry (IHC)

Microarray sections were placed in a pressure cooker containing 1× EDTA retrieval solution and boiled for 30 min. After cooling to room temperature, the slides were washed three times with 1× PBST buffer, 5 min per wash. Endogenous peroxidase activity was blocked with 3% H_2_O_2_ for 5 min, followed by incubation with 5% serum for 15 min. Subsequently, the slides were incubated with specific primary antibodies at 4 °C overnight, then subjected to 3,3′-diaminobenzidine (DAB) staining, hematoxylin counterstaining, and mounting. The results were scored based on the percentage of positive cells (0: 0%, 1: 0%–25%, 2: 26%–49%, 3: 50%–74%, 4: 75%–100%) and staining intensity (0: no staining, 1: weak, 2: moderate, 3: strong). The final evaluation criteria for IHC results were as follows: 0: negative, 1 to 4: positive +, 5 to 8: positive ++, 9 to 12: positive +++.

### Cell culture

Human lung cancer cell lines NCI-H1299 and A549 were procured from iCell Bioscience Inc. Cells were cultured in F-12 nutrient medium supplemented with 10% fetal bovine serum (FBS; Biological Industries). They were maintained at 37 °C in a humidified incubator with 5% CO_2_. All cell lines were authenticated by short tandem repeat (STR) profiling and tested negative for *mycoplasma* contamination. All experiments were performed when cells were in the exponential growth phase.

### Lentivirus RNA interference (RNAi) constructs

To knock down human RPL35A, target sequences were designed, synthesized, and cloned into the BR-V108 lentiviral vector, designated as shRPL35A. The corresponding control vector was named shCtrl. The primers used for RPL35A were 5′-CCTATTTCCCATGATTCCTTCATA-3′ and 5′-GTAATACGGTTATCCACGCG-3′. Single-stranded DNA oligonucleotides were synthesized and annealed in a 90 °C water bath for 15 min to form double-stranded DNA. The resulting double-stranded DNA was ligated into a linearized vector and transformed into E.coli competent cells for amplification. After extraction, the ligation products were co-transfected into 293T cells with helper plasmids. At 72 h post-transfection, the supernatant containing lentiviral particles was harvested to obtain the shRNA lentivirus.

For overexpression of RPL35A or SKP2, target sequences were designed, and the corresponding gene fragments were amplified by PCR. These fragments were cloned into linear vectors, transformed into E.coli competent cells for amplification, and subsequently co-transfected into 293T cells with helper plasmids. At 72 h post-transfection, the supernatant containing lentiviral particles was harvested to generate the overexpression lentivirus.

### Quantitative real-time polymerase chain reaction (qRT-PCR)

Total RNA was extracted from cell lines using TRIzol reagent (Sigma-Aldrich). Genomic DNA was removed using gDNA Wiper Mix (Vazyme), and RNA was reverse-transcribed into cDNA using HiScript QRT SuperMix for qPCR (Vazyme). Quantitative real-time PCR (qRT-PCR) was performed with SYBR Green Master Mix (Vazyme) on an ABI PCR System (Applied Biosystems). GAPDH expression served as the internal control, with all target gene expression levels normalized to GAPDH. Relative expression levels were calculated using the 2^-ΔΔCt^ method. All procedures were carried out following the manufacturers’ instructions. Primer sequences are provided in Table S1.

### Western blot analysis

Cells were washed once with ice-cold PBS and lysed in Western and IP cell lysis buffer. Protein concentrations were determined using a BCA Protein Assay Kit (HyClone-Pierce). Lysates were separated by SDS-PAGE and transferred to PVDF membranes. Membranes were blocked for 1 h, then incubated overnight at 4°C with the following primary antibodies:RPL35A (Abcam, Rabbit, ab241070, 1:2000), MYC (CST, Rabbit, 5605, 1:1000), SKP2 (PTG, Rabbit, 15010-1-AP, 1:2000), GAPDH (Bioworld, Rabbit, AP0063, 1:3000), PKM2 (Bioss, Mouse, bs-0102M, 1:1000), HK2 (Proteintech, Rabbit, 66974-1-Ig, 1:3000), GLUT1 (Bioss, Rabbit, bs-4855R, 1:1000), ADH4 (Abcam, Rabbit, ab137077, 1:2000), ALDOC (Proteintech, Rabbit, 14884-1-AP, 1:5000), and Histone H3 (CST, Rabbit, 4499S, 1:2000). After three TBST washes, membranes were incubated for 1 h at room temperature with species-matched secondary antibodies:Goat Anti-Rabbit,(Beyotime, A0208, 1:3000)and Goat Anti-Mouse(Beyotime, A0216, 1:3000). Proteins were visualized using Immobilon Western Chemiluminescent HRP Substrate (Millipore) on an AI600 imager (GE Healthcare). All experiments were performed in triplicate with representative blots shown.

### Co-immunoprecipitation

Cells were washed twice with ice-cold PBS and lysed in ice-cold IP lysis buffer for 10 min. Lysates were centrifuged at 13,000*g* for 10 min at 4 °C, and supernatants were collected. Total protein concentration was determined. For immunoprecipitation, lysates containing 1.0 to 1.2 mg of total protein were incubated overnight at 4 °C with gentle rocking after adding the target-specific antibody. Protein A/G beads (20 μl) were then added to the antibody-antigen complexes and incubated for 2 h at 4 °C with rotation. Beads were washed three times with ice-cold IP buffer, and bound proteins were eluted by boiling in 5× SDS loading buffer. Eluted proteins were resolved by SDS-PAGE for subsequent Western blot analysis.

### Cell counting assay

Cells were seeded in 96-well plates at densities of 1500–2500 cells per well and cultured at 37 °C with 5% CO_2_ atmosphere. Starting on day 2, cell proliferation was monitored daily for five consecutive days using the Celigo Imaging Cytometry System (Nexcelom Bioscience, Lawrence). Fluorescence intensity and cell counts were quantified, and proliferation curves were generated from statistically analyzed data.

### EDU staining assay

Cells were pulse-labeled with five-ethynyl-2′-deoxyuridine (EdU; 10 μM) for 2 h at 37°C, then fixed and permeabilized. Incorporated EdU was detected using the Click-iT EdU HCS Assay Kit (Thermo Fisher Scientific) according to manufacturer's protocols. Fluorescence signals were quantified and imaged using an epifluorescence microscope system.

### Apoptosis assay

Apoptosis was quantified using an Annexin V-APC Detection Kit (Yeasen Biotechnology). After treatment, cells were harvested, washed twice with PBS, and resuspended in 200 μl 1× binding buffer. Cell suspensions were incubated with 10 μl Annexin V-APC for 15 min at room temperature (RT) in the dark. Binding buffer (400–800 μl) was added to adjust sample volume based on cell density. Samples were analyzed within 1 h using a Guava easyCyte flow cytometer (MilliporeSigma) with FlowJo software.

### Wound-healing assay

Cells were seeded in 96-well plates (5 × 10^4^ cells/well) and cultured overnight to >90% confluence. The medium was replaced with low-serum medium (0.5–2% FBS), and a uniform scratch was generated in each well using a sterile 200-μl pipette tip. After washing with serum-free medium to remove debris, fresh low-serum medium was added. Initial images (t = 0) were captured using phase-contrast microscopy (4× objective). Plates were incubated at 37 °C/5% CO_2_, and migration was monitored at predetermined intervals. Wound closure was quantified by measuring residual scratch area using ImageJ software (v1.53t), and the migration rate was calculated using the corresponding formula.

### Transwell migration assay

Transwell chambers (Corning) were used for this assay. 100 μl of serum-free medium was added to the upper chamber and incubated for 1 h. After removing the serum-free medium, 600 μl of medium with 30% FBS was added to the lower chamber, and 100 μl of cell suspension containing 100,000 cells was added to the upper chamber. The upper chamber was then placed in the lower chamber with 30% FBS medium and incubated for 24 h. Non-migrated cells were removed with a cotton swab, and the upper chamber was transferred to a new lower chamber with 400 μl of staining solution. The chamber was immersed in the staining solution for 5 min, and the migrated cells were stained, photographed, and imaged using an inverted microscope (Olympus IX73).

### Measurements of glucose, lactic acid, and ATP levels

Cells were seeded in 96-well plates (100 μl medium/well) and cultured overnight. For glucose quantification, 5 × 10^6^ cells were harvested, washed with PBS, and resuspended in 1 ml distilled water. Cell suspensions were lysed by ultrasonication (3 × 10 s pulses, 30% amplitude, ice bath), boiled for 10 min, and cooled on ice. Lysates were centrifuged for 10 min at 4 °C. Supernatants were analyzed for glucose content using a Glucose Assay Kit (Solarbio, Beijing, China) according to the manufacturer's protocol. Absorbance was measured at 505 nm using a microplate reader.

Lactate quantification was performed using a Lactate Assay Kit (Solarbio). Cells were lysed in 1 ml of ice-cold lysis buffer (provided in kit) by ultrasonication (3 × 10 s pulses at 30% amplitude, ice bath). Lysates were centrifuged for 10 min at 4°C. A total of 800 μl supernatant was mixed with 150 μl enzyme working solution, incubated at 37 °C for 10 min, and absorbance was measured at 570 nm using a microplate reader.

ATP quantification was performed using an ATP Assay Kit (Solarbio). Cells were lysed in 1 ml ice-cold extraction buffer (6% perchloric acid) by ultrasonication (3 × 10 s pulses at 30% amplitude, ice bath). Lysates were centrifuged for 10 min at 4°C. Supernatants (800 μl) were deproteinized with 500 μl chloroform (vortexed 30 s), centrifuged for 3 min (4°C), and the aqueous phase was collected. Neutralized samples were combined with luciferin-luciferase reagent, and luminescence was measured immediately using a GloMax Discover Microplate Luminometer (Promega).

### Measurement of extracellular acidification rate (ECAR) and oxygen consumption rate (OCR)

Cells were seeded in 96-well plates at a density of 5 × 10ˆ5 cells per well the day before measurement. For ECAR measurement, cellular mitochondrial function and glycolytic capacity were assessed using the acidification detection fluorescent probe.

Cells were seeded in 96-well plates at 5 × 10^5^ cells/well and cultured overnight. Extracellular acidification rate (ECAR) was measured using the P61 fluorescent probe (Extracellular Acidification Rate Assay Kit, BestBio) to assess glycolytic flux. Oxygen consumption rate (OCR) was determined using the R01 fluorescent probe (Oxygen Consumption Rate Assay Kit, BestBio). Following manufacturer protocols, fluorescence was recorded using a microplate reader. ECAR and OCR values were derived from the linear slope of kinetic traces after blank subtraction.

### Affymetrix microarray analysis

Microarray experiments were performed by Shanghai Yibeirui Biomedical Science and Technology Co, Ltd. Total RNA was extracted from cells according to the manufacturer's protocol. First-strand and second-strand cDNAs were synthesized *via* reverse transcription, followed by the synthesis and purification of complementary RNA (cRNA) using the 3′ IVT Plus Kit (Affymetrix, USA). The concentration of cRNA was determined using a spectrophotometer, and labeled cRNA was selected for hybridization. The labeled cRNA was hybridized to the microarray chip, and scanning results were automatically acquired using an Affymetrix Scanner 3000.

### Firefly luciferase & Renilla luciferase assay

Dual-luciferase reporter assay. Transfection complexes were prepared by mixing 20 μl Opti-MEM with 0.1 μg experimental plasmid (control [NC], MYC, SKP2-WT, or SKP2-MUT), 0.01 μg pRL-TK Renilla luciferase plasmid (internal control), and 0.3 μl Lipofectamine 3000 (0.8 mg/ml; Invitrogen). After 15 min incubation at RT, complexes were added to cells in complete medium. Cells were harvested 48 h post-transfection. Luciferase activity was measured using the Dual-Luciferase Reporter Assay System (Promega). Lysates were transferred to 96-well plates, mixed with 75 μl Dual-Glo Luciferase Reagent, and incubated 10 min. Firefly luminescence (reporter) was recorded. After adding 75 μl Dual-Glo Stop & Glo Reagent (10 min incubation), Renilla luminescence (normalization control) was measured.

### Chromatin immunoprecipitation (CHIP)

Chromatin immunoprecipitation (ChIP) was performed using the SimpleChIP Enzymatic Chromatin IP Kit (CST #9003) per manufacturer's protocol. Briefly, 4 × 10^6^ cells were crosslinked with 1% formaldehyde (10 min, RT), quenched with 125 mM glycine, and lysed. Chromatin was digested with micrococcal nuclease (37 °C, 20 min), then sonicated (VirTis Virsonic 100 Ultrasonic Homogenizer/Sonicator; 3 × 20 s pulses at 35% amplitude, 4 °C) to shear DNA to 200 to 500 bp. Immunoprecipitation used:Anti-MYC (Boster, BM4042; rabbit) and Normal Rabbit IgG (CST #2729; negative control). Precipitated DNA was purified and analyzed by qPCR with SYBR Green Master Mix. Primer sequences:SKP2 Promoter Region 1 F: 5′-CACACCCACAATTCAGGAAGAG-3′, R:5′-GACGTGCTACAAGGTGGCA-3′; SKP2 Promoter Region 2 F:5′-ACATTTCCCAGTCAGCCGTAG-3′, R:5′-TCCTTCCCTT GCAGCTTTACC-3′.

### Xenograft transplantation *in vivo*

Four-week-old female BALB/c nude mice (body weight: 16–18 g) were obtained from Cavens Beagle Model Animal Research Co., Ltd (Suzhou, China). All animal experiments were approved by the Ethics Committee of the First Hospital of Shanxi Medical University and were conducted in strict accordance with the EU Directive 2010/63/EU for the protection of animals used for scientific purposes. Mice were housed under specific pathogen-free (SPF) conditions in a controlled environment (temperature: 25 ± 2 °C; 12 h light/dark cycle) with *ad libitum* access to a standard diet and autoclaved water. Bedding was changed once or twice weekly. All procedures were performed following relevant institutional guidelines and regulations.

Ten nude mice were randomly assigned to two groups (n = 5 per group): the shCtrl group (control) and the shRPL35A group (knockdown). A subcutaneous xenograft model was established by injecting A549 cell suspension (1 × 10^7^ cells) into the right flank of each mouse. Tumor dimensions were measured using vernier calipers and and volumes were calculated every 6 days. Mouse body weight was monitored weekly as a measure of overall health. After 30 days, the mice were euthanized, and the tumors were excised, weighed, and photographed. For subsequent analysis, tumor tissues were either fixed in 4% paraformaldehyde for 24 to 48 h for histology or snap-frozen in liquid nitrogen and stored at −80 °C for molecular analysis.

### Statistical analysis

All data were presented as the mean ± standard deviation (SD) and were analyzed using GraphPad Prism 6.0 software. Statistical differences between groups were evaluated using the unpaired Student’s *t* test, with a *p*-value < 0.05 considered statistically significant. The Mann-Whitney U test and Spearman’s correlation analysis were applied as appropriate to assess relationships between two groups. Each experiment was independently repeated three times. When the ANOVA indicated a significant difference, *post hoc* multiple comparisons were performed using Tukey's honestly significant difference (HSD) test. A *p*-value of less than 0.05 was considered statistically significant.

## Data availability

All data are contained within the manuscript.

## Supporting information

This article contains supporting information.

## Conflict of interest

The authors declare that they do not have any conflicts of interest with the content of this article.

## Figures and Tables

**Figure S2:**
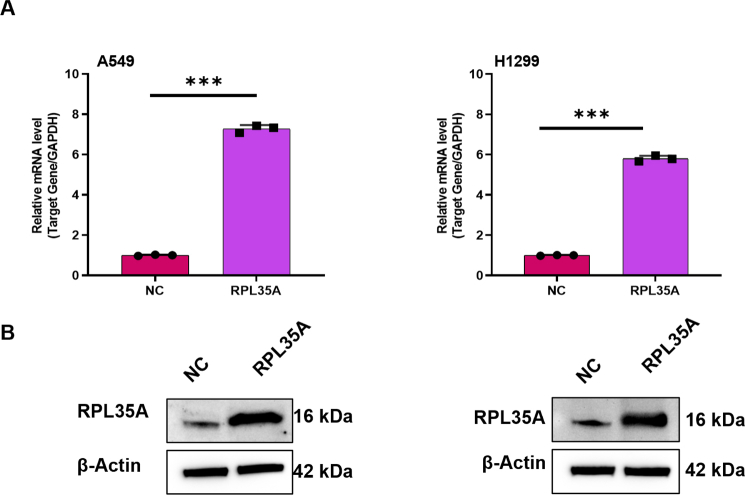


**Figure S4:**