# Supplementary material for: RPL35A drives aerobic glycolysis and tumorigenesis by facilitating MYC-mediated SKP2 transcription
Source: J Biol Chem. 2025 Nov 13;302(1):110944. doi: 10.1016/j.jbc.2025.110944 (PMC12816858; doi:10.1016/j.jbc.2025.110944)
Supplement: Supplementary Table [file mmc1.doc]

**Supplementary Table 1. Primers designed for qRT-PCR**

| Gene | Forward primer sequence (5’-3’) | Reverse primer sequence (5’-3’) |
| --- | --- | --- |
| GAPDH | TGACTTCAACAGCGACACCCA | CACCCTGTTGCTGTAGCCAAA |
| GAPDH | TGACTTCAACAGCGACACCCA | CATGGGCCACGATCCTCTTTA |
| RPL35A | GAAGGTGTTTACGCCCGAGAT | CGAGTTACTTTTCCCCAGATGAC |
| MYC | CATACATCCTGTCCGTCCAAG | CAAGAGTTCCGTAGCTGTTCAA |
| SKP2 | ATAGAAGTGTCCACCCTCCACG | CACCCAGAAAGGTTAAGTCGC |
| SKP2 | CACACCCACAATTCAGGAAGAG | GACGTGCTACAAGGTGGCA |
| SKP2 | ACATTTCCCAGTCAGCCGTAG | TCCTTCCCTTGCAGCTTTACC |
| RRAS2 | GAGGCATCAGCAAAGATTAGG | TTGGTTCTGGTGAAGGAGGA |
| CDC7 | CAGGGGTATGGATTCTAGCACTC | TGTCCTGGAGGTGTTTGAACG |
| RPS18 | CATAGAGGGCTGCGTCACTT | CTTCTTGGACACACCCACGG |
| RPS2 | GTGGATGCCCGTCACCAAGTT | TGATTCCTTAATAGGCAGGGAGAAGA |
| EIF3C | CAGAAAATGAGGGCGAGGAC | TAGATGTGGCAGAGGATGGC |
| BRAF | GAAGAATACACCAGCAAG | AAAGGCTAGAAGAGGAAGAA |
| MCM2 | CCAATGGCTTCCCTGTCTT | TCATCGGTCAGTTCCCCTAC |
| FANCD2 | GGCTTGACAGAGTTGTGGATG | TAGGATCTCAGGTAGGCTGGTG |
