# Supplementary material for: RPL35A drives aerobic glycolysis and tumorigenesis by facilitating MYC-mediated SKP2 transcription
Source: J Biol Chem. 2025 Nov 13;302(1):110944. doi: 10.1016/j.jbc.2025.110944 (PMC12816858; doi:10.1016/j.jbc.2025.110944)
Supplement: Supplementary legends [file mmc2.doc]

Figure S1 Knockdown efficiency of RPL35A was examined by qPCR and WB

Figure S2 Overexpression efficiency of RPL35A was examined by qPCR and WB

Figure S3 Some downstream genes were screened by Affymetrix microarray analysis after knockdown of RPL35A.

Figure S4 RPL35A and SKP2 were examined after RPL35A knockdown and SKP2 overexpression by qPCR and WB.
